# The Home Environments of Infants of Mothers with Early, Remitted Clinical Depression and No Depression during the First Two Years Postpartum

**DOI:** 10.3390/children10091471

**Published:** 2023-08-29

**Authors:** Lauren M. Henry, Nanmathi Manian, Gianluca Esposito, Marc H. Bornstein

**Affiliations:** 1 Eunice Kennedy Shriver National Institute of Child Health and Human Development, Bethesda, MD 20892, USA; lauren.henry@nih.gov; 2 Westat, Rockville, MD 20850, USA; nanmathimanian@westat.com; 3 Department of Psychology, University of Maryland, Baltimore County, Baltimore, MD 21250, USA; 4 Department of Psychology and Cognitive Science, University of Trento, 38068 Rovereto, Italy; gesposito79@gmail.com

**Keywords:** maternal clinical depression, infancy, home environment, early experience effects

## Abstract

The current study examines stability, continuity, and group and gender differences in the home environments of infants of mothers with early, remitted clinical depression and no postpartum depression, overcoming methodological variations in the extant literature. Fifty-five mothers diagnosed with clinical depression (major or minor depression, dysthymia, or depressive disorder not otherwise specified) at 5 months and fully remitted by 15 and 24 months, and 132 mothers with no postpartum depression (*M*_age_ = 32.47; 69.7% European American) completed the Home Observation for Measurement of the Environment (HOME) Inventory Infant/Toddler version when their infants were 15 and 24 months old. No differences in stability estimates of the HOME scales were found between the groups. In terms of continuity, controlling for maternal education and infant birth order, HOME responsivity, involvement, and total score decreased, while HOME acceptance increased between 15 and 24 months in the full sample. There were no effects of group or gender. Results may point to the home environment as a key protective factor for infants of mothers with early, remitted clinical depression, or findings may suggest improved maternal parenting cognitions and practices following remission.

## 1. Introduction

Postpartum maternal depression is a condition with public health significance; in the United States, approximately 13% of new mothers experience depression symptoms [1,2], and between 7% and 17% are diagnosed with a major depressive episode after childbirth [3,4]. Global prevalence rates are even more striking; in low- and middle-income countries, between 15% and 57% of women suffer from postpartum depression [4,5]. Despite an urgent need, fewer than 27% of women with postpartum depression symptoms receive treatment, and only 6% of women receive adequate treatment (defined as six weeks using antidepressants continuously or engaging in established and accepted psychotherapy) [6]. By 36 months postpartum, approximately 16% of mothers with depression achieve remission [7].

The weight of the statistics describing the prevalence, treatment, and course of maternal depression compounds when considering consequences beyond the individual [8,9,10,11]. Particularly relevant to the current study is the impact of maternal depression on child development [10]. Exposure to maternal depression alters developmental trajectories across multiple domains of child well-being, including psychological health [12], physical growth [13], cognition and language [14], and social functioning [15]. Although genetics and other exposures and experiences play roles [12], the passing of risk from mother to child in the context of depression occurs, in significant part, by way of parenting and the home environment.

As described in Bronfenbrenner’s bioecological systems theory [16], individual development is shaped by multiple, interdependent environmental systems that differ in their developmental impact. Parent, home, and family are the most proximal and arguably most influential systems [16]. These influences are especially salient during infancy, a critical developmental period during which children rely on parents to meet their basic needs and children spend the majority of their time in the home environment [17,18]. The quality of the home environment in the first three years of life is related to later cognitive development [19], and individual studies have demonstrated the significance of the home environment to academic achievement, social skills, and psychological symptoms [20,21].

Critically, parental psychopathology impacts the quality of the home environment. For example, Goodman and Brumley [22] found that homes of mothers with depression scored lower on measures of environmental quality than homes of mothers with no psychiatric diagnoses. One shortcoming of this body of work is that the majority of studies has measured maternal depression using self-report questionnaires of symptoms [23,24,25,26]. Mothers who self-report depression symptoms in the postpartum period may not ultimately meet the diagnostic criteria for clinical depression, experiencing transitory symptoms instead [27,28,29]. Problematically, self-reports fail to distinguish profiles of mothers with “postpartum blues” (i.e., time-limited depression symptoms [30]) from profiles of mothers with clinical depression [31,32]. A second shortcoming of this body of work is that associations between maternal depression and the home environment during the first two years of life have been examined only in very few studies. Instead, researchers have recruited families directly after birth [33], during the preschool years [24,25], or much later [34]. A third shortcoming is that few, if any, studies have examined the isolated early effects of clinical maternal depression (i.e., later remitted) on the home environment during infancy. Similarly, stability (consistency in individual differences across time) and continuity (consistency in group means across time) have rarely been represented in depression research [35]. Notwithstanding a large literature on the maladaptive consequences of maternal depression for child development [12], some research has supported selective child recovery following remission (that is, in some children and on some outcomes [36,37]). Of note, it is likely that the aforementioned designs were implemented to appropriately test study-specific hypotheses. However, our understanding of the impact of maternal depression on infancy has been limited by neglecting key issues. The current study was designed to advance the literature on maternal depression and the home environment by addressing these shortcomings. 

We examined the home environments of mothers with early, remitted clinical depression (i.e., depressed at 5 months but fully remitted at 15 and 24 months) and mothers with no depression during the first two years postpartum in the context of a rigorous methodological framework. We assessed maternal depression at 5 months to isolate clinical depression, compared to the postpartum blues and other transient disturbances associated with being a new parent [30]. Mothers participated in a clinical interview with trained mental health professionals to determine depressed versus nondepressed group membership. We assessed the home environment at 15 and 24 months; during this time, parenting behaviors and practices stabilize [38], and infants experience rapid development in domains that allow them to explore and actively engage with the environment [39]. To assess the home environment, we used 4 scales of the Home Observation for Measurement of the Environment (HOME) Inventory Infant/Toddler version [40]: responsivity, acceptance, organization, and involvement. We recruited families of middle socioeconomic status (SES) in the two groups to obviate confounding depression with low SES, and additional maternal (e.g., age, ethnicity, marital status, education, verbal intelligence) and infant (e.g., age, gender, birth order) sociodemographic characteristics were evaluated and controlled in the analyses as appropriate. Careful controls are important, especially considering research that supports HOME scale estimates as moderately related to SES and maternal education [41].

Based on previous research [41], we expected low to moderate individual-level consistency (stability) across time with magnitude varying by scale such that responsivity and acceptance would be the least stable, and organization and involvement would be the most stable. We expected to observe group-level consistency (continuity) across time for all HOME scales. Considering the dearth of research on stability and continuity of the home environment among infants of mothers with early, remitted clinical depression, our examination of group differences in stability and continuity was exploratory. Based on a limited body of research, we surmised that infants of mothers with early (i.e., 5 months) clinical depression would have later home environments (i.e., at 15 and 24 months when depression had fully remitted) characterized by lower levels of physical and social stimulation and support than infants of mothers with no postpartum depression. We also explored gender differences in infant home environments.

## 2. Materials and Methods

The study was conducted according to guidelines in the Declaration of Helsinki, with written parental informed consent obtained prior to data collection. All procedures involving human subjects were approved by the Institutional Review Board at the National Institutes of Health (NIH) Clinical Center, Eunice Kennedy Shriver National Institute of Child Health and Human Development Institutional Review Board under Protocol 02-CH-0278 (NIH Clinical Trials Identifier: NCT00044174).

### 2.1. Participants

New mothers (older than 20 years old) in the Washington, DC, metropolitan area were recruited via mass mailings, women’s groups, and newspaper advertisements and by consulting birth records. Recruitment materials targeting mothers with depression provided information about the study, including its longitudinal nature and focus on postpartum depression and child development.

Across the two groups (i.e., early, remitted clinical depression and no postpartum depression), the mothers averaged *M* = 32.47 years old (*SD* = 4.52). The majority (90.8%) of the mothers were married. Furthermore, 69.7% of the mothers identified as non-Hispanic European American, 13.0% as African American, 9.2% as Latin American, 4.9% as Asian American, and 3.2% as mixed or other ethnicity. With regard to education, 20.5% of the mothers reported partial college or less, 36.8% had completed college, and 42.7% had completed university graduate programs.

The infants averaged *M* = 5.17 months old (*SD* = 0.26), *M* = 14.99 months (*SD* = 0.36), and *M* = 24.34 months (*SD* = 0.42) at the three assessments. Fifty-one percent of the infants in the early, remitted clinical depression group and 58% of infants in the no postpartum depression group were boys. The majority (61.6%) of the infants were firstborn. All infants were full term and healthy. No infants had known genetic disorders or birth complications.

### 2.2. Measures

#### 2.2.1. Maternal Depression

Maternal depression symptoms were evaluated using the Beck Depression Inventory-II (BDI-II) [42]. The BDI-II is brief self-report measure of the presence and degree of depressive symptoms in the previous 2 weeks, consistent with the *Diagnostic and Statistical Manual of Mental Disorders* (4th ed.; DSM-IV) [43]. The BDI-II consists of 21 items scored on 4-point scales ranging from 0 to 3 [42].

Maternal depression diagnoses were determined using the Structured Clinical Interview for DSM-IV Axis I Disorders—Non-patient research version (SCID-I) [44]. The SCID-I is a semi-structured interview designed to facilitate DSM-IV Axis I diagnoses [44]. Certified mental health professionals familiar with the DSM-IV classification and diagnostic criteria were trained to administer the SCID-I. In the current study, the diagnostic flow of the interview consisted of the SCID-I Overview, Mood Episodes, Psychotic Screening, and Modules D to J (Mood Disorders, Substance Use, Anxiety, Somatoform, Eating, Adjustment, and Optional Disorders). At 5 months, the definition of “current” episode was changed to “within the lifetime of the child”. At 15 and 24 months, the questions pertained to “current” symptoms, as well as symptoms since the time of the last interview. Of note, the DSM-IV provides diagnostic criteria for major and minor depression and dysthymia. Briefly, five or more depression symptoms (including depressed mood or anhedonia) for 2 weeks or more causing significant distress or impairment are required for a diagnosis of major depression. Two to four depression symptoms are required for a diagnosis of minor depression. Manic and hypomanic episodes should not be present for either major or minor depression. Dysthymia is characterized by the predominance of a depressed mood for at least 2 years with no interruption of symptoms for 2 months or more. In addition to depressed mood, two or more symptoms of dysthymia causing significant distress or impairment are required for a diagnosis of dysthymia, and manic, hypomanic, or mixed episodes or major depressive disorder should not be present during the first 2 years. Depressive disorder not otherwise specified (DD-NOS) is defined as a depressed mood with clinically significant impairments which do not meet the criteria for the duration or severity of major or minor depression or dysthymia [43]. Four pilot SCID-I interviews were audio recorded and scored by two certified mental health professionals; the diagnostic consensus was 100%.

#### 2.2.2. The Home Environment

The home environment was assessed at 15 and 24 months using the Home Observation for Measurement of the Environment (HOME) Inventory Infant/Toddler version [40]. The HOME is a semi-structured home observation and interview with the primary caregiver, which is applicable for children from birth to age 3. The HOME was developed to measure stimulation and support available to children in their homes, in terms of both quality and quantity. The HOME was originally designed in the 1960s and has been used extensively worldwide and in normal and at-risk populations [40]. Ninety percent agreement between observers and internal consistency estimates between 0.44 and 0.89 were found in the first study assessing the psychometric properties of the HOME [41]. The general conclusions reached from several additional studies have been that the interobserver agreement is ≥0.80, and the internal consistency of the scales ranges from 0.30 to 0.80 [45]. Four of the six HOME scales and a summed total score were used for the current study, which consisted of 31 items scored as *Yes/No* in the following domains: responsivity of parent (e.g., parent spontaneously reports/praises child’s qualities), acceptance of child’s behavior (e.g., parent does not scold child during visit), organization of the environment (e.g., child has special place for toys), and parental involvement with the child (e.g., parent talks to child while doing household work). The items were totaled such that higher scores indicated a more enriched environment.

#### 2.2.3. Maternal Verbal Intelligence and Controls

Maternal verbal intelligence was assessed at 5 months using the Peabody Picture Vocabulary Test—Revised (PPVT-R) [46]. For each of 175 vocabulary words presented, the mother was asked to indicate the meaning of the word by selecting one of four pictures. Standard scores were obtained (*M* = 100, *SD* = 15, range = 40–160). In a sample of 828 adults ranging from 19 to 40 years of age, the median split-half reliability coefficient was 0.82 [46].

Mothers also rated comfort and typicality of the home visits on 3-point rating scales ranging from 1 (*very comfortable or typical*) to 3 (*not comfortable or typical*). On average, mothers reported that they were comfortable (*M* = 1.57, *SD* = 0.51). On average, mothers rated infant behaviors (*M* = 1.27, *SD* = 0.50) and their own behaviors (*M* = 1.26, *SD* = 0.46) as typical. No differences were found between groups regarding maternal comfort or infant and maternal behavior typicality: *ts* (118) = −0.59, *p* = 0.65, 1.76, *p* = 0.08, and 0.88, *p* = 0.38, respectively. Infants were awake for approximately 99.22% of the home visit. No group difference was found in infant wakefulness: *t* (118) = 0.49, *p* = 0.69.

### 2.3. Procedures

The BDI-II was mailed to 536 mothers between 1 and 5 months postpartum [42]. Four hundred sixty-one mothers returned the BDI-II, and 316 mothers with low (<7) and high scores (>12) were invited to complete the SCID-I at 5 months postpartum. Mothers diagnosed with depression (major depression, minor depression, dysthymia, or DD-NOS) were selected into the clinically depressed group (*n* = 121). Mothers without depression diagnoses were selected into the nondepressed group (*n* = 195). 

The SCID-I was administered again at 15 and 24 months postpartum [44]. The majority of mothers diagnosed as clinically depressed at 5 months had fully remitted (i.e., at least 2 months without significant depression symptoms) by 15 and 24 months. In the present analyses, only mothers who were diagnosed as clinically depressed at 5 months and had fully remitted by 15 and 24 months were included. Fifty-five mothers with early and remitted clinical depression (henceforth referred to as the “depressed group”) and 132 mothers with no postpartum clinical depression (henceforth referred to as the “nondepressed group”) provided some or all data. Of the 55 mothers in the depressed group, 39 mothers were diagnosed with major depression (70.9%), 12 mothers with minor depression (21.8%), and 4 mothers with DD-NOS (7.5%) at 5 months.

A 45- to 90-min home visit with the child and caregiver is indicated for the HOME Inventory Infant/Toddler version [40], including a semi-structured observation and interview with goals of minimizing intrusiveness and promoting typical family interactions. Data collection for the current study was structured accordingly. Mothers and infants were visited in their homes for 60 min of naturalistic ongoing interaction. Only the mother, infant, and a female researcher were present. Mothers were asked to behave in their usual manner. The home visit was scheduled for a time when the infant was awake and alert, and the mother was responsible for her infant’s care. Various routine mother–infant activities occurred during the visit, including feeding, diapering, bathing, and playing.

### 2.4. Preliminary Analyses and Data Analytic Plan

#### 2.4.1. Missing Data Analysis

Available sample sizes were examined for all variables. Several variables were missing from the HOME Inventory—ranging from *n* = 5 (2.67%, for HOME Involvement at 15 months) to *n* = 16 (8.55% for HOME Responsivity at 15 months). Five dyads were missing the same scale for both 15 and 24 months (two in the depressed group and three in nondepressed group). Whether the missing data for the HOME Inventory were differentially distributed was examined across the depressed and nondepressed groups. Chi-square analysis showed that the pattern of missing data did not differ between the groups (*χ*^2^s ranged from 0.15, *p* = 0.70, to 3.57, *p* = 0.06). To maximize the sample size and the power, missing data were imputed with the series mean separately for the depressed and nondepressed groups and for those who were missing HOME variables at either 15 or 24 months but not both. Thus, the final sample sizes for all subsequent analyses were *n* = 55 for the depressed group and *n* = 132 for the nondepressed group. See Consort Flow Diagram in Supplementary Materials. Of note, we conducted analyses with the original data (with missing values for the HOME scales) and the data with imputed values. The results were the same. For ease of presentation and interpretation, we present the results with the imputed data.

#### 2.4.2. Preliminary Analyses

The distributions of all variables were examined for normality, outliers, and influential cases by examining indices of non-normality and Q-Q plots [47]. There were no problems with normality or outliers in the distributions of variables. An a priori power analysis was conducted to determine whether the sample size provided sufficient power to detect medium-sized effects in a 2 × 2 (age × depression group or age × gender) repeated-measures ANOVA design. With an effect size of 0.15 for within-subjects effects and 0.25 for between-subjects effects [48], α = 0.05, and *N* = 187, the power estimates ranged from 0.92–0.99 for main effects and interactions, indicating sufficient power to detect medium and large effects. The power estimates to detect a correlation of 0.30 with α = 0.05 in the full sample (*N* = 187) and in the nondepressed group (*n* = 132) were 0.99 and 0.97, respectively, indicating strong power to detect medium and large correlations. The power to detect a correlation of 0.30 within the depressed group (*n* = 55) was 0.71, and for a correlation of 0.50, the power was 0.99, indicating marginal power to detect a medium effect and strong power to detect a large effect.

There were no differences in maternal age between the depressed (*M* = 32.68, *SD* = 4.71) and nondepressed (*M* = 32.38, *SD* = 4.45) groups, *F* (1, 183) = 0.17, *p* = 0.68. There were no differences in infant age between the depressed (*M* = 159.05 days, *SD* = 9.41) and nondepressed (*M* = 156.71 days, *SD* = 6.80) groups, *F* (1, 185) = 2.91, *p* = 0.08. There were no differences in marital status between the groups (married_depressed_ = 85.7%, married_nondepressed_ = 93.0%, *χ*^2^ (1, 185) = 2.50, *p* = 0.11) or ethnicity between the groups, *χ*^2^ (4, 185) = 2.20, *p* = 0.70. Maternal education, coded into three categories, differed between the groups, *χ*^2^ (2, 184) = 5.93, *p* = 0.04; the depressed group had higher percentages of “less than college-educated” mothers (28.6%) than the nondepressed group (17.1%) and lower percentages of college graduates (30.4%) than the nondepressed group (48.1%). Further analysis revealed that maternal education was significantly associated with one of the outcome variables, HOME Acceptance at 24 months, *F* (2, 167) = 4.70, *p* = 0.01; hence, maternal education was used as a covariate. The two groups did not differ in PPVT-R: *F* (1, 183) = 0.51, *p* = 0.47. The percentages of infants who were firstborn also differed between the groups; 47.0% of infants in the depressed group and 14.6% of infants in the nondepressed group were firstborns: *χ*^2^(1, 185) = 6.10, *p* = 0.01. Further analysis revealed that firstborn status was significantly associated with HOME Involvement at 24 months: *F*(1, 171) = 8.78, *p* = 0.003; hence, firstborn status was used as a covariate.

#### 2.4.3. Data Analytic Plan

We first present descriptive statistics and stability estimates across ages 15 to 24 months for the HOME by group. Stability was assessed by Pearson’s partial correlation coefficients, controlling for maternal education and infant birth order. We then report continuity across infant age and the effects of group and gender, as well as two-way interactions (age × group, age × gender) for the HOME administered at 15 and 24 months, employing repeated-measures analysis of covariance (RM-ANCOVA), controlling for maternal education and infant birth order. Infant age was treated as a within-subjects variable, and depression group status (clinically depressed vs. nondepressed) was treated as a between-subjects variable.

## 3. Results

### 3.1. Descriptive Statistics and Stability

Table 1 presents descriptive statistics and stability (zero-order Pearson’s correlation coefficients) across time for all scales by group. The scales showed low to moderate stability, with *r* values ranging from 0.22 to 0.43. Based on Fisher’s z-transformations, no significant differences emerged in stability estimates between HOME scales or between groups for any of the HOME scales.

### 3.2. Infant Age, Group, and Gender

Table 2 presents RM-ANCOVA results of depression group and gender from 15 to 24 months, with maternal education and infant birth order as covariates, for all HOME scales. 

#### 3.2.1. Responsivity Scale at 15 and 24 Months

Controlling for maternal education and infant birth order, the RM-ANCOVA revealed a main effect of infant age: HOME responsivity decreased from 15 to 24 months (*Estimated marginal means (EMM)* = 9.79, *SE* = 0.08; *EMM* = 7.40, *SE* = 0.10, respectively). We found no other main effects or interactions.

#### 3.2.2. Acceptance Scale at 15 and 24 Months

Controlling for maternal education and infant birth order, the RM-ANCOVA revealed a main effect of infant age: HOME acceptance increased from 15 to 24 months (*EMM* = 6.25, *SE* = 0.10; *EMM* = 7.46, *SE* = 0.12, respectively). We found no other main effects or interactions.

#### 3.2.3. Organization Scale at 15 and 24 Months

Controlling for maternal education and infant birth order, the RM-ANCOVA revealed no main effects or interactions.

#### 3.2.4. Involvement Scale at 15 and 24 Months

Controlling for maternal education and infant birth order, the RM-ANCOVA revealed a main effect of infant age: HOME Involvement decreased from 15 to 24 months (*EMM* = 4.70, *SE* = 0.09; *EMM* = 3.81, *SE* = 0.08, respectively). We found no other main effects or interactions.

#### 3.2.5. Total Score at 15 and 24 Months

Controlling for maternal education and infant birth order, the RM-ANCOVA revealed a main effect of infant age: HOME total score decreased from 15 to 24 months (*EMM* = 25.99, *SE* = 0.18; *EMM* = 24.07, *SE* = 0.20, respectively). We found no other main effects or interactions.

## 4. Discussion

Maternal postpartum depression is prevalent [4,5] and woefully undertreated [6]. Research has documented the maladaptive effects of maternal depression on child development [12], but the sustained impact of early, remitted clinical depression on the infant’s home environment (another critical determinant of healthy child development) is unclear. In the current study, we used a rigorous methodological framework to investigate the home environments of infants of mothers with early clinical depression which had later remitted (i.e., depressed at 5 months and fully remitted at 15 and 24 months) and infants of mothers with no depression during the first 2 years postpartum. We examined stability, continuity, and group and gender differences of the home environments of these two groups.

In terms of stability (individual-level consistency), the estimates were low to moderate in magnitude, but statistically significant. HOME scales were low to moderately stable across the clinically depressed and nondepressed groups, and there were no significant group differences in stability. Also, no significant differences in stability estimates across scales emerged. With regard to continuity (group-level consistency), the Responsivity, Involvement, and Total Score scales decreased between 15 and 24 months, while the Acceptance scale increased between 15 and 24 months. There was no change in the Organization scale between 15 and 24 months. No differences in continuity estimates emerged between the clinically depressed and nondepressed groups. Also, no differences in gender emerged.

The results generally support findings of a somewhat dynamic home environment during the first years of life, regardless of maternal diagnostic profiles [41,49]. Estimates of the HOME scales may vary across families and across time due to any number of factors, including child maturation and capabilities and interventions [50,51,52]. Next, despite previous research documenting deficits in the home environments of mothers with depression relative mothers without psychiatric diagnoses, and contrary to our expectation, in the current study we found no differences in the home environments of mothers with early clinical depression which had later remitted (i.e., depressed at 5 months and fully remitted at 15 and 24 months) and mothers with no postpartum depression. The results may reflect the robustness of the home environment to early clinical depression such that the home environment may serve as a protective factor for infants of mothers who have experienced remission. Alternatively, the results may point to improvements in parenting cognitions and practices (here, greater responsivity, acceptance, and involvement and better organization of the environment) following the remission of early clinical depression [36]. Additional research is needed to elucidate these potential effects. A strong home environment may be one factor contributing to the selective recovery of some infants from the negative effects of early maternal depression [36,37], and future research should explore this explanation directly. Regardless, it is clear that early identification of, and evidence-based intervention for, early maternal depression to ensure remission is critical to supporting the healthy development of mother and child during the postpartum period and beyond [12].

The current study takes advantage of a methodologically rigorous design, including depression diagnoses through clinical interviews with trained mental health professionals (allowing us to distinguish clinical depression from the postpartum blues), careful controls reducing bias in our estimates (maternal age, ethnicity, marital status, education, and verbal intelligence; infant age, gender, and birth order; recruitment of middle-SES families), and the investigation of underresearched, yet important, factors influencing the sequalae of maternal depression, including remission, stability, and continuity. Still, our findings must be interpreted in light of several limitations. Our sample consisted of mothers who were European American and middle-SES with full-term, healthy infants. Accordingly, our results are generalizable only to similar populations, and future research should explore the current research questions in diverse samples [34]. In addition, research supports the HOME Inventory as being a reliable and valid measure of the home environment, with the semi-structured home observation and interview format providing enhanced ecological validity relative to parent reports. Still, the HOME provides only a “snapshot” of the home environment on a single day for a single hour in the lives of children and families. With technological advances, future research may benefit from replicating the current findings using a measure of the home environment with better ecological validity at more frequent assessments. For example, systems such as the Electronically Activated Recorder [53] and the Language ENvironment Analysis [54] facilitate the collection of audio recordings from families as they go about their daily lives [55], and machine learning may facilitate analysis of large volumes of data [56].

## 5. Conclusions

We examined the home environments of infants of mothers with early clinical depression which had later remitted (i.e., depressed at 5 months and fully remitted at 15 and 24 months) and infants of mothers with no depression during the first two years postpartum. Taken together, the findings may support the home environment as potentially robust to the otherwise maladaptive effects of early, remitted maternal depression. Additionally, the findings may suggest improvements in the home environment following remission from depression.

## Figures and Tables

**Table 1:** Descriptive statistics and stability (partial correlations) across time for HOME inventory scales.

	Descriptive Statistics				Stability	
	15 Months		24 Months			
HOME Inventory	*M*	*SD*	*M*	*SD*	*r*	*r* ^2^
Responsivity Scale						
Depressed	9.81	0.76	7.31	1.27	0.32 *	0.10
Nondepressed	9.78	1.05	7.54	1.10	0.22 *	0.05
Acceptance Scale						
Depressed	6.20	1.22	7.18	1.67	0.22	0.05
Nondepressed	6.20	1.27	7.69	1.34	0.38 ***	0.14
Organization Scale						
Depressed	5.27	0.72	5.38	0.72	0.23	0.05
Nondepressed	5.28	0.70	5.41	0.70	0.23 **	0.05
Involvement Scale						
Depressed	4.65	1.03	3.84	1.06	0.28 *	0.08
Nondepressed	4.72	1.05	3.76	0.93	0.39 ***	0.15
Total						
Depressed	25.89	1.79	23.71	2.66	0.43 **	0.19
Nondepressed	25.94	2.36	24.36	2.34	0.43 ***	0.19

*Note. n* = 55 for depressed group and *n* = 132 for nondepressed group. * *p* ≤ 0.05. ** *p* ≤ 0.01. *** *p* ≤ 0.001.

**Table 2:** RM-ANCOVA results for HOME inventory scales for depressed/nondepressed groups with maternal education and infant birth order as covariates.

	Responsivity Scale 15–24 Months		Acceptance Scale 15–24 Months		Organization Scale 15–24 Months		Involvement Scale 15–24 Months		Total Score 15–24 Months	
	*F* (1, 178)	η^2^_p_	*F* (1, 178)	η^2^_p_	*F* (1, 178)	η^2^_p_	*F* (1, 178)	η^2^_p_	*F* (1, 178)	η^2^_p_
Infant Age (Time)	211.89 ***	0.54	35.83 ***	0.17	2.07	0.01	49.78 ***	0.22	46.89 ***	0.21
Group	0.53	0.003	1.41	0.008	0.005	0.000	0.30	0.002	0.48	0.003
Gender	0.20	0.001	0.43	0.002	3.52	0.02	0.29	0.002	0.21	0.001
Age × Group	1.27	0.007	3.35	0.02	0.04	0.000	0.40	0.002	2.06	0.01
Age × Gender	0.53	0.003	0.75	0.004	0.99	0.006	3.80	0.02	0.02	0.000

*Note*. *n* = 55 for depressed group and *n* = 132 for nondepressed group. All analyses control for maternal education and infant birth order. *** *p* ≤ 0.001.

## Data Availability

The study data can be made available to authorized parties upon application to the second and corresponding author.

## Supplementary Materials

The following supporting information can be downloaded at: https://www.mdpi.com/article/10.3390/children10091471/s1, CONSORT 2010 Flow Diagram.
