# Supplementary figures and images for: The Home Environments of Infants of Mothers with Early, Remitted Clinical Depression and No Depression during the First Two Years Postpartum
Source: Children (Basel). 2023 Aug 29;10(9):1471. doi: 10.3390/children10091471 (PMC10528664; doi:10.3390/children10091471)

## CONSORT 2010 Flow Diagram

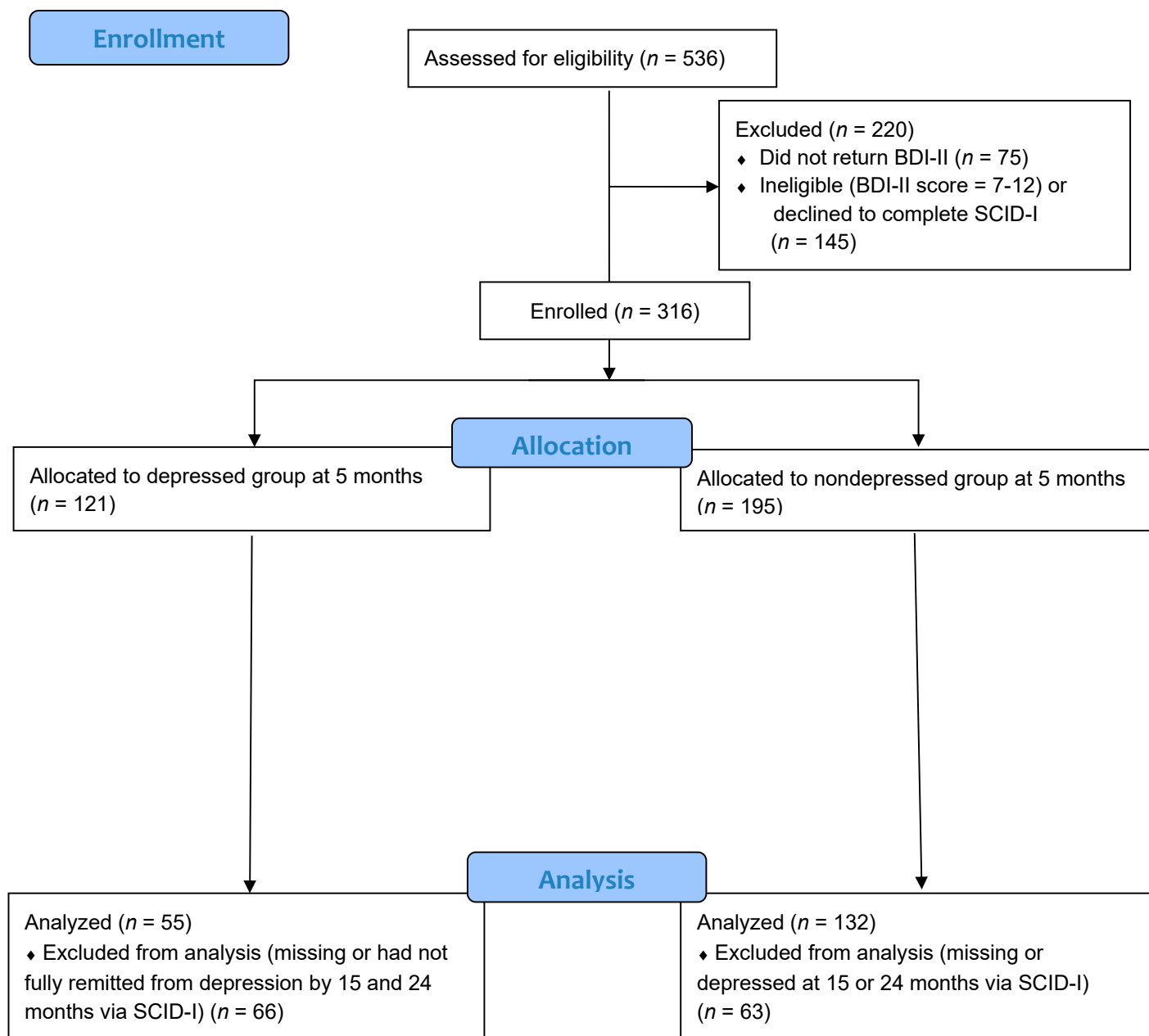

Supplement: Supplementary file 1 [file children-10-01471-s001.zip › children-2479349-supplementary.pdf]
